# The mitochondrial genome of *Tetrahymena rostrata*

**DOI:** 10.1080/23802359.2019.1693919

**Published:** 2019-12-09

**Authors:** A. Watt, R. Haites, N. Young, H. Billman-Jacobe

**Affiliations:** Faculty of Veterinary and Agricultural Sciences, Melbourne Veterinary School, University of Melbourne, Parkville, Australia

**Keywords:** *Deroceras reticulatum*, Ciliophora, *Tetrahymena rostrata*, mitogenomics

## Abstract

*Tetrahymena rostrata* is a ciliated protist which can parasitize the gray field slug, *Deroceras reticulatum.* Here, we report the sequence of the mitochondrial genome (mt genome) of a strain of *T. rostrata* that was isolated from the egg of *D. reticulatum.* Whole cell genomic DNA was sequenced using Illumina^®^ MiSeq and the mitochondrial DNA sequence reads were extracted and assembled. The resulting 47,235 bp assembly contained rRNAs, tRNAs, and 45 protein coding DNA sequences of which 21 encoded proteins of unknown function. Phylogenetic analysis showed *T. rostrata* clustered with *Tetrahymena thermophila*, *Tetrahymena pigmentosa*, *Tetrahymena pyriformis*, *Tetrahymena paravorax*, and *Tetrahymena malaccensis.*

*Tetrahymena rostrata* (Kahl, 1927) is a ciliate which lives in soil litter or as a parasite of some terrestrial mollusks (Brooks 1968). It is able to kill *Deroceras reticulatum* slugs and has been considered as a biological control agent for pest mollusks. There is limited sequence information for *Tetrahymena* species. The mitochondrial DNA sequence of *T. rostrata* was determined in order to make phylogenetic comparisons with other members of this genus.

*Tetrahymena rostrata* strain TRAUS was isolated from an egg laid by a laboratory-reared *D. reticulatum* (Melbourne, Australia, 37°47′45.9′′S; 144°57′30.7′′E) and has been deposited in the American Type Culture Collection, Virginia, USA (PTA-126056). *Tetrahymena rostrata* was grown in axenic culture and genomic DNA was extracted from whole cells. A sequencing library, prepared using the Illumina^®^ TruSeq kit, was enriched using KAPA Taq DNA polymerase (Kapa Biosystems) and sequenced using Illumina^®^ MiSeq (Illumina, San Diego, CA). The raw data were filtered using the mitochondrial genome (mt genome) of *Tetrahymena pigmentosa* (Moradian et al. 2007) as a reference and the reads were *de novo* assembled with Unicycler version 0.4.7 (Wick et al. 2017) followed by gap filling. The sequence was annotated using Geneious Prime version 1.3 (Biomatters Ltd., Auckland, New Zealand) (Kearse et al. 2012) with reference to other *Tetrahymena* mt genomes (Burger et al. 2000; Brunk et al. 2003; Moradian et al. 2007) and was deposited in GenBank (accession number MN025427).

The *T. rostrata* mt genome was a 47,235 bp linear DNA and had a GC% of 21.8%. The gene organization was the same as other *Tetrahymena* mt genomes (Burger et al. 2000; Brunk et al. 2003; Moradian et al. 2007). The genome has two rRNAs (large and small subunits, each consisting of two units), seven tRNAs, and 23 genes encoding proteins of predicted function (ribosomal proteins L2, L14, L16, S3, S12, S13, S14, and S19; NADH dehydrogenase subunit proteins Nad1, Nad1a, Nad2, Nad3, Nad4, Nad4L, Nad5, Nad6, Nad7, and Nad9; ATP synthase F0 subunit 9, cytochrome oxidase 1 and 2, apocytochrome b and heme maturase). This species shared the genes of unknown function, *ymf*56, 57, 59–61, 63–78, also identified in other *Tetrahymena* (Burger et al. 2000). The small ribosomal RNA sequence was located centrally and the large ribosomal RNA subunits occurred as terminal inverted repeats which were flanked by tRNAs and telomeric repeats. The amino acid coding regions of *T. rostrata* and other Hymenostomatida mt genomes were translated and concatenated for comparison using MAFFT version 7.388 (Katoh and Standley 2013) and the Bayesian phylogenetic inference was performed using a Markov chain Monte Carlo (MCMC) analysis in MrBayes version 3.2.6 (https://github.com/NBISweden/MrBayes) using a 11,000,000 MCMC generation chain length with consensus trees generated using the 50% majority rule criterion and the final 90% of trees generated by Bayesian inference after a burn-in of 100,000 generations. *T. rostrata* clustered with other members of the *Tetrahymena* genus although *Tetrahymena paravorax* appears to be an outgroup (Figure 1).

**Figure 1.**
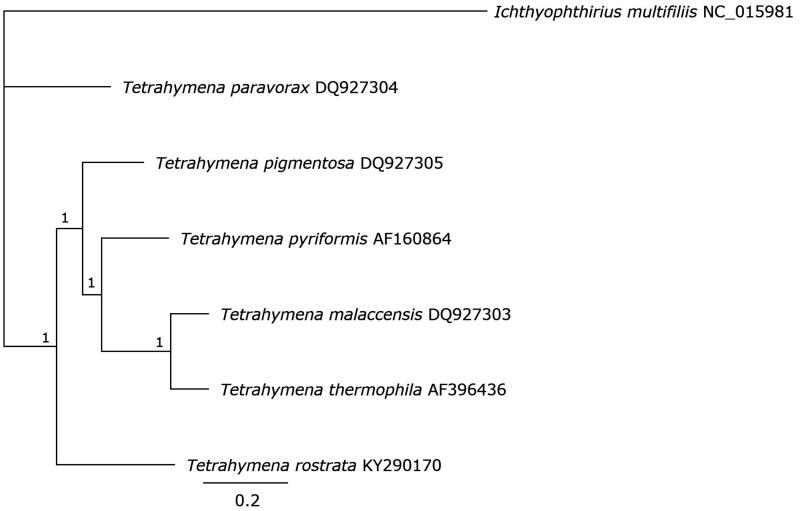
MrBayes tree of the translated, concatenated amino acid coding regions of the mt genomes of *T. rostrata* TRAUS, and other Hymenostomatida available in GenBank.
